# It Is Time to Consider Glucagon-Like Peptide-1 Receptor Agonists for the Treatment of Type 2 Diabetes in Youth

**DOI:** 10.3389/fendo.2019.00738

**Published:** 2019-10-29

**Authors:** Megan M. Oberle, Aaron S. Kelly

**Affiliations:** ^1^Department of Pediatrics, University of Minnesota, Minneapolis, MN, United States; ^2^Center for Pediatric Obesity Medicine, University of Minnesota Medical School, Minneapolis, MN, United States

**Keywords:** GLP-1 receptor agonists, pedatrics, type 2 diabetes, obesity, weight management, adolescents

Prior to the SEARCH for Diabetes in Youth, a large multicenter study that described the prevalence of diabetes in the pediatric population, type 2 diabetes (T2DM) was mainly thought of as a disease of adulthood. However, SEARCH highlighted the startling rise in the prevalence of T2DM in children, adolescents, and young adults in the United States, citing a nearly 30% increase between 2001 and 2009 (1). Although much was known about the etiology of T2DM and time-course of treatment failure in adults, relatively little was known about the increasing pediatric population with T2DM until the Treatment Options for T2DM for Adolescents and Youth (TODAY) Study in 2012. The TODAY study highlighted that youth with T2DM have earlier and more rapid deterioration of β-cell function than adults despite treatment with two anti-diabetic agents (metformin and rosiglitazone) (2, 3). The median time to treatment failure (defined as defined as HgbA1c ≥ 8% over 6 months, inability to wean insulin within three months of its initiation, or need to begin insulin therapy) was approximately 1 year for metformin monotherapy, metformin plus rosiglitazone, or metformin plus intensive lifestyle modification (2). Additionally, youth with T2DM were more likely to have kidney disease, dyslipidemia, retinopathy, and/or hypertension at baseline and experience earlier incidence of these co-morbidities than adults (2). Despite the poor outcomes with standard treatments and the evidence suggesting that the pathophysiology of T2DM in youth is particularly aggressive, metformin and insulin were the only FDA-approved treatments for youth with T2DM in the U.S. until recently (4–7). Unfortunately, neither metformin nor insulin target the underlying β-cell dysfunction, meaningfully reduce excess adiposity, or reasonably delay the need to initiate insulin to maintain glycemic control (8).

In the TODAY study and in clinical practice, failure of metformin and lifestyle modification often required the initiation of insulin therapy earlier than desired as a means of rapidly improving glycemic control to delay the long-term sequelae of hyperglycemia (nephropathy, neuropathy) (3, 5). The International Society for Pediatric and Adolescent Diabetes (ISPAD) Clinical Practice Guidelines recommend starting basal insulin if a patient fails to reach HbA1c <6.5% after 3–4 months of metformin monotherapy and adding meal time or correction rapid-acting insulin if the goal HbA1c is not achieved (5). However, insulin-associated weight gain can be a barrier to adherence and negatively influence motivation to lose weight in youth with T2DM (9). The recommendation to start insulin therapy following metformin failure in youth is in contrast to the guidelines for adults with T2DM, which recommend avoidance of insulin therapy because of the associated weight gain (10). In the management guidelines for adults with T2DM, insulin or sulfonylureas are only added to the regimen if other medications (GLP-1 RAs, DPP-IV inhibitors and SGLT2 inhibitors) fail to achieve goal HbA1c as both insulin and sulfonylureas promote weight gain, which can worsen insulin resistance and overall health (10).

With the recent FDA-approval of the daily injectable glucagon-like peptide-1 receptor agonist (GLP-1 RA) liraglutide, there is now another safe and effective option for treatment of T2DM in youth that promotes both weight loss and improves glycemic control. GLP-1 RAs mimic endogenous GLP-1 hormone action, thereby stimulating endogenous insulin secretion, decreasing glucagon levels, possibly delaying gastric emptying, and decreasing appetite (11). GLP-1RAs may also improve β-cell function, as shown in animal models, a property that could be especially relevant to youth, secondary to the more progressive nature of B-cell deterioration as compared to adults with T2DM (12–14). Although there are multiple forms of GLP-1 RAs on the market for adults, liraglutide is the only medication of this class approved for youth down to age 10 years. Phase 3 trials of other GLP-1 RAs as well as other classes of medication commonly used in adults (SGLT2 inhibitors and DPP-4 inhibitors) in youth with T2DM are currently ongoing so additional options may be available in the next few years (15).

The Evaluation of Liraglutide in Pediatrics With Diabetes (Ellipse) trial generated the data from which the decision for FDA-approval of liraglutide for youth down to age 10 years was based (16). In the trial, liraglutide doses up to 1.8 mg daily were found to decrease the mean HbA1c levels by 1.3 percentage points over approximately 1 year and improve fasting glucose in youth (aged 10 to 17 years) with T2DM and overweight/obesity compared to placebo (16). The Ellipse trial did not find a significant reduction in body weight between the liraglutide and placebo groups; mean body weight decreased in both groups at week 26 (−2.3 kg with liraglutide and −0.99 kg with placebo) (16). Similarly, a smaller (*n* = 28) randomized, double-blind, placebo-controlled trial found that liraglutide decreased HbA1c over 5 weeks compared to placebo, but did not result in a significant weight loss over this relatively short treatment period (17). Although no GLP-1 RA trials have been conducted in youth with T2DM with a primary focus on weight reduction, GLP-1 RAs have been found to promote weight loss in youth with obesity without diabetes (18–20). For example, a randomized, double-blind, placebo-controlled trial evaluating the safety and tolerability of liraglutide in pediatric participants (age 7–11 years) reported that doses up to 3.0 mg daily significantly reduced BMI z-score by −0.3 kg/m^2^ compared to the placebo group (20). In adults, GLP-1 RAs were associated with weight loss in patients with overweight or obesity with and without type 2 diabetes, as well as reduction in adverse cardiovascular outcomes and reduced progression of diabetes-related nephropathy (21–26).

Liraglutide was found to be both safe and well-tolerated in youth with obesity and T2DM with escalating daily doses, up to 1.8 mg daily (T2DM treatment dose) (16, 17, 27), or 3.0 mg daily (obesity treatment dose) (20, 28). In all studies evaluating liraglutide in youth, transient GI side effects (nausea, vomiting, diarrhea, and abdominal pain), similar to those experienced with metformin, were the most commonly reported though these effects can be mitigated with slow up-titration (16, 17, 20, 28, 29). Hypoglycemia can also occur with GLP-1 RAs; however, this risk is lower than with insulin and other anti-diabetic medication (25, 30, 31). No severe hypoglycemia events, defined as plasma glucose <56 mg/dL, were reported in the five trials of liraglutide in youth (16, 17, 20, 28). In the randomized, double-blind, placebo-controlled trial evaluating the safety and tolerability of liraglutide in pediatric participants without diabetes, mild asymptomatic hypoglycemia, ranging from 62 to 70 mg/dL, was reported in 25% of participants in the liraglutide-treated group (20). While pancreatitis has been associated with GLP-1 RA use in some adult studies, a meta-analysis of clinical trials did not demonstrate a link between GLP-1 RA treatment and pancreatitis in adults with T2DM (32, 33). Incidence of pancreatitis was not reported in the pediatrics trials of liraglutide (16, 17, 20, 28). In general, safety and tolerability profiles in the pediatric trials were comparable to adult studies; and consistent with adult studies, body weight and gender appear to influence liraglutide pharmokinetics (16, 17, 20, 27, 28).

Based on results of the SEARCH and TODAY studies published nearly 5 years ago, clinicians treating youth with T2DM have known that treatment with metformin alone will likely be insufficient to decrease diabetes progression and reduce insulin requirements. Furthermore, it is now well-established that adding insulin therapy in an effort to decrease hyperglycemia and prevent retinopathy, neuropathy, and nephropathy will likely exacerbate weight gain and may discourage youth from actively engaging in lifestyle management. The recent FDA-approval of liraglutide presents a safe option to pediatric endocrinologists to avoid weight gain while effectively managing glycemic control. Now is the time to consider GLP-1 RAs as a preferred treatment for youth with T2DM, along with metformin and lifestyle modification.

## Author Contributions

All authors listed have made a substantial, direct and intellectual contribution to the work, and approved it for publication.

### Conflict of Interest

MO serves as site PI for study through Vivus Pharmaceuticals. AK receives research support (drug/placebo) from Astra Zeneca Pharmaceuticals and serves as a consultant for Novo Nordisk, Orexigen, and Vivus Pharmaceuticals but does not accept personal or professional income for these activities.
